# Infant Feeding, and Its Influence on Life; or, the Causes and Prevention of Infant Mortality

**Published:** 1861-09

**Authors:** 


### NORTH AMERICAN

### MEDICO-CHIRURGICAL REVIEW.

SEPTEMBER, 1861.

### Analytical and Critical Reviews.

ART. I.—*Infant Feeding, and its Influence on Life; or, the Causes and Prevention of Infant Mortality.* By C. H. F. ROUTH, M.D., M.R.C.P.E., M.R.C.S., Physician to the Samaritan Free Hospital for Women and Children, etc., etc. 12mo., pp. 379. London, 1860.

WHEN an infant is provided with a full supply of milk from the breast of a healthy mother or of a suitable nurse, its diet, for at least the first eight or ten months of its existence, need give but little or no concern. If permitted to follow its proper instinct, it will be well nourished, and run little danger of injury from improper, deficient, or over feeding. The case is very different, however, when, from any cause, the infant has been deprived, either from its birth or shortly afterward, of its natural aliment. The question as to how and upon what it is to be fed becomes then one of grave importance. In relation to it serious misconceptions are constantly made by the community at large, while the most erroneous views are entertained by even the best-informed members of the medical profession. The consequence has been the infliction of a large amount of suffering and disease upon the infant population of our own and other countries, and the augmentation to a very great extent of its mortality. Nor is the mischief resulting from the want of an acquaintance with the proper feeding of infants confined to such as are unfortunately deprived of the maternal breast, and in consequence brought up by the hand, as it is termed; in some degree, it extends to nearly every infant at the period of weaning—the period when it is to be fed entirely upon other nutriment than that which had been previously supplied by the mother's milk. The evil consequences to the child of errors in diet immediately after weaning may not, perhaps, be so promptly manifested, nor to the same extent, as are those resulting from an injudicious system of feeding, in the case of infants brought up entirely by hand; yet, from their more insidious character, and the greater number by which, in this country at least, they are incurred, they become of equal if not greater importance. In every point of view, indeed, the subject of "infant feeding" is one replete with the deepest interest to all who feel that the cause of true philanthropy and the best interests of every community are to be promoted by shielding as much as possible the offspring of both rich and poor from all avoidable suffering, by reducing the amount of disease and of mortality liable to occur during the first months of their existence, and by insuring to them in after-life symmetrical and healthful frames, and a fair chance for the enjoyment of a prolonged and useful existence.

The treatise of Dr. Routh, while it recommends itself to our consideration from the nature of its subject, will be found, upon examination, to be one of the most able exponents of it, in its several departments and bearings, that has yet appeared. The author has evidently studied the subject of infant feeding thoroughly and carefully, and with great industry has collected and compared all the facts and statistics upon record adapted to throw light upon it. His general conclusions appear to be perfectly reliable, and are throughout of a directly practical application.

Some portions of the work had appeared as separate papers in the medical periodicals of England. They have, however, been all carefully revised, and in the volume before us are presented in a more matured and complete form.

*Part first* of the treatise is devoted to a consideration of the *mortality of infants* in the city and country, and at the different periods of the year; with an examination of some of the more prominent of the causes which influence the mortality among foundlings especially.

All authorities are in accord as to the very great mortality which takes place among the inmates of foundling hospitals; and so far as reliable statistics have been obtained, they bear out this conclusion. The cause of the mortality among foundlings has been very generally ascribed to the fact of their being brought up by the hand and without breast-milk. This Dr. Routh has shown, however, to be an error. His investigations have convinced him that there is no one law of mortality common to all foundling hospitals, or even to the same institution in different years. On the contrary, the causes of the mortality in these institutions are numerous and diversified—some of them being, doubtless, endemic to particular hospitals.

There is always a much greater ratio of deaths during infancy in cities and large towns than in strictly rural districts. Although we are in possession of no extended series of statistics in which the mortality of infants in town and country is positively indicated, yet, so far as the data within our reach have been analyzed and compared, they show very clearly the baneful influence exerted upon the health and life of the infant by the atmosphere of cities. In Ireland, while the general mortality of children under one year of age is 14·7 per cent. in the towns, it is only 8·8 in the rural districts, while that of children under five years of age is 8·1 in the towns and 4·3 in the country. The same fact, according to Dr. Routh, is true also in respect to England, and will, we believe, hold good everywhere. If, then, the mortality is so much greater among all town-reared children of tender age than it is among country children, how is it with respect especially to foundlings?

"Unfortunately, however," remarks Dr. Routh, "after long inquiry, I do not find this distinction made in any work on foundlings which I have seen; nevertheless, I have attempted to adduce it from some figures given in the general statistics of foundlings in France, published by authority of the government. But here, as in the case of infants in general, the data being insufficient, I am unable to obtain more than an approximative result. The relative mortality, however, between town hospital foundlings and those placed in the country thus comes out more strikingly than we might have supposed. Thus, in five years, out of 52,883 *town hospital foundlings*, the mortality was 72·2 per cent. Out of 122,110 *country hospital foundlings*, the mortality was 11·5 per cent. This conclusion proves that foundling hospitals, if established at all, should always be placed in the country."

The mortality among infants, including foundlings, is shown by all writers to be greatest during the first twelve months of existence. According to Quetelet, nearly *twenty-five* per cent. of all children born die within the first year after birth. During the first year, also, the mortality is the greatest in the first month, and gradually decreases onward to the twelfth.

There is a curious circumstance pointed out by Dr. Routh, which is, that in Ireland the deaths among infants generally, during the first month after birth greatly exceed those among infants of lying-in hospitals, being about 31·6 per cent. for the civic and 22·2 for the rural districts, while in the Irish lying-in hospitals, taking the deaths of infants for ten years, from 1831 to 1841, the percentage is only 6·1.

Among the other causes of infant mortality, which more particularly affect foundlings, Dr. Routh enumerates impure air, arising, in part, from defective ventilation, but more generally, and to a much greater extent, from the mere aggregation of a number of infants in the same wards. It is this also which renders the inmates of such institutions more obnoxious to contagious and infectious diseases. The peculiar hospital atmosphere, which is generated in the best planned and most carefully conducted foundling hospitals and orphan asylums, when their wards are crowded with healthy and diseased inmates, is liable to give rise also to certain maladies which, though they may be met with without these institutions, in over-crowded, confined, and filthy dwellings and neighborhoods, are still, to a great extent, the special endemics of the former.

The milk of a strange nurse is of itself often a source of mortality in foundlings and quite young orphans. This may result from the unadaptedness of the nurse's milk, from a variety of causes, to the infant attempted to be nourished by it, its unnutritive or unwholesome qualities, or because it is not supplied to the nursling sufficiently often, or in adequate quantity, when the nurse has an infant of her own to suckle, the interests of which being, in many cases, attended to at the expense of the nursling.

"So far, then," says Dr. Routh, "we have certain causes in operation, which are not exclusively confined to foundling hospitals, which may explain some of the mortality, viz.: Residence in town will account for from 5·07 to 6·1 per cent. for children under one year old. During the first month, if the number of inmates of hospitals die only in proportion to the number which die out of them in civic districts, the mortality may be as high as 33 per cent. Of this amount, under the most favorable circumstances, 6·1 per cent. must take place in the first month. At least from 16·6 to 34·1 per cent., or the mean of 25·3 per cent., should be put down for the effect of contagious diseases. To absence of the *mother's* breast-milk, 10 per cent. per annum may at least be referred: in all, 47 per cent., at least for children under five, from causes not peculiar to foundlings—a large percentage to be deduced from their usual mortality. It should also not be lost sight of that, as the mortality of children is generally greatest in the earlier years, and as, in a given population of foundlings, there is a higher percentage of younger children, so, necessarily, the whole percentage mortality must be greater."

The causes of mortality to which foundlings are especially exposed are numerous. Among these is set down the effects of removal. The injury which infants suffer from this source are due in part, no doubt, to want of care, defective nourishment, and partly to exposure during the passage, when this is of any distance. Another cause of mortality especially affecting foundlings is the influence of the seasons. The cold, wet, and changeable seasons being those most prejudicial to infantile life. In England, Dr. Routh makes the greatest number of deaths among young children in the public institutions to take place in the spring, the least number in autumn, while it is about the same in winter and summer. Thus, the deaths at all seasons being 100, 30·8 will occur in spring, 27·1 in summer and winter, while in autumn they will be only 14·3.

We know of no statistics from which can be determined, with any degree of accuracy, the influence of the seasons upon the mortality of infants in the public institutions in this country. Judging from the mortuary tables of some of the Eastern, Middle, and Western States, we should set down the largest mortality to the summer months; next to these would be the latter winter and early spring months; while the most friendly to infant life would be the months of May, October, November, and December.

The influence of a continued recumbent position and defect of exercise is unquestionably one of the causes of the large mortality in foundling hospitals. A child requires good food and pure air, but these are not enough to insure its health. It must be moved about, have its position frequently changed, and be carried out often into the open air and sunlight. It must be not merely well and properly nourished, but its entire hygienic management must be carefully attended to also.

The want of breast-milk is another of the especial causes of mortality among foundlings and orphans generally. Dr. Routh believes it to be, however, a less influential cause than is generally supposed. He thinks this is rendered evident by a reference to the actual number of deaths attributed to want of breast-milk, occurring among a general population, irrespective altogether of foundling institutions. From the Registrar-General's tables for London, taking the six years, 1849 to 1854, inclusive, it will be found that out of 473,865 births, 15,241, or 3·2 per cent., died from want of breast-milk, in its widest sense; or, out of 73,227 deaths from all causes, of children under one year of age, 20·8 per cent. might be referred to deprivation of the natural food.

The deductions of Dr. Routh, from the facts and statistics he has presented, are thus recapitulated:—

"1. That for the ages of one year and under five, the mortality, even under ordinary circumstances, is in towns nearly double what it is in the country; but this difference in the mortality, according to residence, is nearly seven times as great for foundlings: therefore, foundlings should never be maintained in towns.

"2. That in Ireland, while it is doubtless very high in the first month, for those under one year it is only 30 per cent. in towns, and 22 per cent. in country; the worst mortality with foundlings being 50 per cent.

"3. That traveling in fair seasons is not dangerous to foundlings.

"4. That the mortality is greatest in spring, and least in autumn, with children in public institutions.

"5. That a chief cause in the mortality of foundlings is want of exercise, and the abuse of the recumbent position.

"6. That the want of breast-milk will only account for a mortality of 3·4 per cent. additional.

"7. That a depraved hospital atmosphere and certain endemic contagious disorders are the chief causes of the mortality in foundling hospitals."

The *second part* of Dr. Routh's treatise is devoted to the subject of *wet nursing*.

The advantages which result to both parent and child from the use, during infancy, of breast-milk, especially that furnished by a healthy mother, as the sole aliment, is insisted upon in the outset. The more ample the supply of breast-milk, and the more exclusively the infant is confined to it during the first twelve months after birth, the more perfect and symmetrical, it has been proved, is the child developed, the more vigorous and robust its constitution, and the least predisposed is it to many of the maladies liable to occur during the early periods of existence.

The mortality of infants who are artificially fed, that is, without the breast, far exceeds that which takes place among such as are nourished entirely by the breast. It is probable, however, that some portion of the mortality among infants reared by the hand is to be attributed rather to the injudicious method of feeding than simply to a deprivation of the mother's milk.

Dr. Routh lays it down as the imperative duty of mothers to suckle their own offspring in every case in which there exists no valid and unavoidable objection to their doing so; and this duty is based, among other reasons, upon the benefits its performance confers upon both child and parent. There is a very great difficulty in procuring an adequate substitute for the infant's natural nurse in the breast of another female, suitable in respect to health, temper, competency, and trustfulness, with a supply of milk, at the same time, sufficient in quantity, of good quality, and adapted in its age to that of the infant to be nourished by it.

It is believed that the case will seldom occur when a mother need relinquish entirely the pleasing duty of suckling her infant. If she be weak, her weakness may be, in great measure, removed by a proper diet and regimen, and, if indicated, a suitable course of tonic remedies. Even when the secretion of milk is defective, it is possible in many, if not in all cases, to increase its quantity and improve its quality by a proper course of treatment.

Dr. Routh passes in brief review some of the reasons usually urged by mothers for declining the office of nurse to their own infants. First, the existence of some specific disease, such as scrofula, phthisis, mania, cancer, syphilis, and other dangerous hereditary diseases. The author admits, in the great majority of cases, the validity of this reason. Mothers so affected are unquestionably justifiable in refusing to nurse their infants. In the case, however, of syphilis and scrofula, both of which affections are often very amenable to treatment, he believes that the objection will not hold good. This is doubtless true in regard to a syphilitic taint in the mother. In such case the child is, also, most generally affected, and the treatment upon which the mother must be put for her cure will also be the one adapted to restore it to health. It should be remembered, that by putting the diseased infant to the breast of a wet nurse there is danger of her becoming contaminated. We differ from Dr. Routh, however, when the question is one of a scrofulous mother or one very decidedly predisposed to the disease. We are convinced that the good of both mother and child demands that the latter should be deprived of the maternal breast.

When it is clearly ascertained that the mother's milk disagrees with the child, it will, Dr. Routh admits, constitute also a legitimate cause for not allowing her to suckle it. Even in such a case, however, change of air and food, in respect to the mother, with wine, proper artificial food, and medicine for the infant, will, after two or three weeks of perseverance, he thinks, frequently remove all source of irritation.

"Those cases, I must say," he remarks, "which have resisted these measures, have occurred either in a very early or a very late period of nursing. In the former instances, the simple substitution of a wet-nurse seldom suffices alone. So much treatment is required besides, that it is often a difficult question to determine how much is due to treatment, and how much to the wet-nurse: still, I must admit that, in a few cases, the salutary effect of the change is very obvious. In the latter periods of suckling, *i.e.* after eight months, weaning often proves as effective as the adoption of another wet-nurse."

The whole of the remarks of Dr. Routh on the important question of the selection of a wet-nurse are pertinent and sound.

The physical qualifications essential for a wet-nurse are set down by him as follows:—

"1. *She should have good milk.*"

"Good human milk has an average specific gravity of 1032, varying from 1030 to 1034. It is always strongly alkaline; this alkalinity it usually retains from five to six days, when it becomes acid. To the taste it is sweet, much more so than cow's milk. When allowed to stand, it will be seen to separate into two portions. The superficial very white substance, known familiarly as *cream*, is chiefly the oil-globules, which, being of a lower specific gravity than the other portions of the milk, rise to the surface. The more transparent subjacent liquid, known popularly by the name of *skim*-milk, when the cream has been removed from its surface, consists of casein, sugar, and salts, held in suspension or solution in a white, opaque liquor. * * * If the subjacent portion, after the cream has separated from it, be kept any time, the sugar contained in it becomes converted into lactic acid, which gradually precipitates the casein as a curd. Rennet, or the mucous membrane of the stomach, and most acids, have the same effect. The fluid which now remains, technically called *whey*, contains still in solution a large quantity of sugar and the salts of milk, which are readily separated by evaporation. When looked at through the microscope, milk is found to consist of a colorless fluid, the *liquor lactis*, in which are floating a number of bodies: (*a.*) *Oil-globules*, similar to those found in all parts of the body. (*b.*) The proper *milk-globules*, smaller in size, varying from $\frac{1}{95000}$ths to $\frac{1}{7000}$ths of an inch. These also appear to be oil-globules, from the fact that they reflect light strongly; but from the difficulty experienced in dissolving them in ether, they are evidently covered with a layer of something else, which surrounds them as a capsule. (*c.*) There are a great multitude of *small granules* or *granulated corpuscles* floating among the milk-globules, most abundant in milk secreted at a very early period. The *liquor lactis* holds in solution the casein, though some observers believe that the external layer of the milk-globules is also made up of casein. * * * There exist, in addition, certain *volatile principles*, which may be obtained by the evaporation or distillation of milk, and to which, in great measure, is probably due the odor of new milk. * * * It may suffice here to make a remark in reference to two of these—the *extractive matters* and the volatile principles of milk. Of the first it may be stated, that most of those peculiar changes in milk which render it detrimental, do not occur so much in the casein, sugar, butter, or salts as in the extractive matters, concerning which, it must be added, in the emphatic words of Lehmann, so aptly used by Becquerel and Vernois, 'we know absolutely nothing.' The same is true in reference to the volatile principles, the nature of which has not yet been determined by chemical inquiry, although several experiments for that object have been made."

"2. *Her hereditary predisposition should be good.*"

Every female in whom there is present an evident predisposition to consumption, or tuberculosis generally, to cancer, mania, and other hereditary affections, as well as every one in whom there exists the slightest taint of syphilis or other similarly communicable disease, should be invariably rejected as a wet-nurse.

"3. *The age of a nurse should not exceed thirty, nor be much under twenty-five years.*"

It is true, Dr. Routh admits, that the milk is not found very much altered, chemically or microscopically, between the ages of fifteen and forty. Still, at the extremities of the scale, the differences are obvious.

"In the very young, the butter, casein, and solid matters generally—excepting the sugar, which exists in diminished quantity—are on the increase. In the older women there is a larger proportion of water and sugar, the amount of butter and casein is diminished, although the latter is still in excess, as compared with the normal condition, from which it may be inferred that the milk of a very young person is less digestible, and therefore less to be recommended for a delicate infant."

"4. *The nurse should not have been confined many months before or after the child's mother.*"

The composition of the milk undergoes a material change as the period of lactation is prolonged; hence the female may supply a milk which will prove injurious to an infant younger or older than her own. The influence exerted by the periods of lactation upon the milk are thus summed up by Becquerel and Rodier:—

"The specific gravity varies much. The proportion of water increases from the fifth to the sixth month, and from the eleventh to the twelfth; it diminishes from the first to the second, and from the eighteenth to the twenty-fourth. The solid matters increase in a marked degree from the first to the third. The sugar decreases during the first month, but increases from the eighth to the tenth month. The butter increases considerably up to the sixth month, and then considerably decreases from the fifth to the sixth, and from the tenth to the eleventh month. The salts undergo a slight increase in quantity from the first to the fifth month, then correspondingly decrease. These changes are really most important to trace, as they are an index as to the substitute which, bearing a proper proportion to the amount and quality of nutritive matter required, is best fitted for a child whom it becomes obligatory to wean, or for whom another diet is imperatively demanded. It must be confessed, however, that the constituents of milk vary so much that it is very difficult readily to estimate its goodness from their present quantity. Its composition varies also, within the limits of health, so much, that we have often no better method of testing it than trial with the child, when, if it agrees with it, we may conclude it is good."

"5. *A wet-nurse of a melancholic temperament should be preferred.*"

This requirement is founded upon the supposition that *brunettes,* whom Dr. Routh supposes to be of a melancholic temperament, give generally a milk richer in solid constituents than that of blondes, to whom he ascribes a sanguineous or scrofulous temperament. We have found good milk furnished by the breasts of females, when young, healthful, and robust, of all varieties of temperament. Females easily excited and given to violent fits of passion, are always, however, to be objected to as wet nurses, as also those of an eminently nervous temperament, who are excitable, fearful, hysterical. In all such it is notorious that the milk is liable to undergo a change, suddenly or slowly, which is particularly injurious to the infant suckled at the breast.

"6. *The wet-nurse should have not only good milk, but it should be in sufficient quantity.*"

During the first year of life, to supply the amount of nutritive matter demanded by the constant and extensive organic changes which then take place, and sufficient materials for the rapid growth and development of the body, requires a large quantity of food; much larger, proportionably, than would suffice an older child or an adult.

When the breast of a suitable wet-nurse cannot be secured, exclusively, for an infant, Dr. Routh recommends that a married woman who is suckling another child be employed to assist the artificial feeding.

The *third part* of the treatise is devoted to a consideration of *the causes of defective assimilation* in children, in reference to the principles of alimentation.

The defect of assimilation observed in infants brought up without the breast, Dr. Routh believes to be the result, not so much of their being thus deprived of a proper supply of good breast-milk, as of injudicious feeding, and general mismanagement.

From an analysis of the Registrar-General's reports, he finds that the mortality among children under one year of age from disease of the nutritive function is 22·3 per cent., while that from the proper diseases of infancy is only 7·1. This striking preponderance in the early months of existence of diseases of the nutritive function—diseases of the abdominal organs, atrophy, marasmus, rickets, etc.—is shown also by a reference to the records of hospitals, etc., as well as by the repeated statements of physicians based upon their own clinical experience in the rounds of private practice.

All those affections of infancy which can be fairly attributed to a defective, impeded, or disordered condition of the formative, reproductive, and nutritive processes, brought on mainly by injudicious feeding and nursing, bad air, and want of cleanliness, Dr. Routh includes under the one general denomination of "*defective assimilation.*" As predisposing causes of this morbid condition he enumerates a hereditary tubercular taint, and debilitating diseases, more especially the sequelæ of the exanthemata.

Defective assimilation presents in its course three distinct stages. In the *first* or *premonitory*, the child may at times appear as in ordinary health and spirits; generally, however, it is peevish and irritable at intervals; the flesh becomes flabby and harsh to the feel; the food taken is often vomited, having a very acid smell; there is loss of appetite and disturbed sleep; constipation may be present, the stools when passed being like clay with white lumps in them. In the *second* or *emaciative* stage, there is an increase of all these symptoms; a more decided irritation of the intestinal canal; more frequent vomiting; with perhaps diarrhœa, the discharges being deep green, very offensive, and so acid as often to excoriate the anus and neighboring parts; there is rapid emaciation; the eye assumes a peculiar bright expression; the child acquires an aged appearance. Sometimes there is no diarrhœa; when, however, stools occur they are loaded with undigested matters. In the *third* or *exhaustive* stage, all the above symptoms are present in an aggravated degree. The child eagerly craves food, nothing seems to satisfy it; but all the food it takes does it no good. Apthæ appear in the mouth, and gradually extend downward into the alimentary canal. There is usually an excessive and unmanageable diarrhœa, the stools being chiefly composed of undigested food. The emaciation is excessive. The child has the appearance of a wrinkled old man; the eyes are unnaturally bright and seem to project from their sockets; it does not sleep, but is constantly whining and crying for food, until it expires in an extreme state of inanition.

When the disease assumes the apthous form, especially in institutions where a large number of children are crowded together, according to Dr. Routh it may acquire an exceedingly malignant and contagious character. It will then often produce, in those to whom it is communicated, an apthous condition not only of the mouth, but also of the conjunctiva and other mucous membranes.

When diarrhœa is absent the disease may run a protracted course of many weeks.

"Sometimes the disease having reached the second stage, or while yet in the first stage, does not pass on to the third; that is, the *primary* assimilation is defective only, but not entirely prevented. *Tuberculosis*, with other developmental disorders, then makes its appearance, generally as *tabes mesenterica*, more rarely as *phthisis*. By far the most common of the diseases it gives rise to is *anœmia*, with more or less of *rachitism*. Painful as these latter complications are to behold, they exhibit, nevertheless, a fortunate phase in the original disease, because, under proper treatment, they are comparatively manageable, whereas defective assimilation, in the third stage, is very seldom if ever cured."

The *post-mortem appearances* are, great emaciation; an almost entire absence of fat; scanty cellular and wasted muscular tissue. When diarrhœa has been present, the entire alimentary canal is beset with red patches and aphthæ from the size of a pin's head to that of a bean, which, after a time, become loaded, more or less, with the *öidium albicans*. Peyer's glands are also much reddened and swollen. Sometimes apthæ are absent, and the mucous membrane, from below the liver, is much reddened, having a coating of bloody and intensely acid mucus. In cases unattended with diarrhœa there is paleness of the intestinal mucous membrane, with considerable enlargement of Peyer's glands. These often project in the form of round patches some three or four lines broad by ten or twelve long, apparently filled with exudation, precisely similar to the appearance they have in cases of Asiatic cholera.

The first and most essential step in the prophylaxis and cure of defective assimilation is a proper attention to the diet of the infant, in respect to the quality and quantity of its food, the manner of feeding, to its cleanliness, its warmth, the purity of the air it breathes, and its hygienic management generally. It is essential to the well-being of the young infant, especially, that it be kept warm, particularly during the times it is fed. It is important, also, that it be made to assume, when in the act of taking food, the semi-erect, which is the natural position.

These principles of treatment are separately discussed by Dr. Routh. In respect to diet, he has shown, from an examination of the aliment of all young animals, that animal food is indispensable to the human infant; this alone furnishing the elements which are required for the proper nourishment, growth, and sustenance, generally, of the body.

The most suitable food in the case of the human infant is without dispute that supplied by the breast of a healthy mother. This contains in proper proportions and beautiful combination the plastic, hydro-carbonaceous, and saline ingredients necessary for the support of life and to meet the exigencies of the system in the early periods of existence. Hence, when pure and taken in sufficient quantity it is capable alone of sustaining the vitality and vigor of the human organism for any length of time.

Dr. Routh advances a proposition of very great importance, in the alimentation of infants and very young children, but which is too often, to a certain extent, lost sight of, if not entirely ignored, by both parents and physicians; it is, that the human being, during the early period of existence, is so constituted anatomically and physiologically, as to be capable of digesting animal food alone.

A full supply of milk from the maternal breast, or that of a healthy and suitable nurse, is all that is required by the infant, so far as food is concerned, for the first year, at least, of its existence. When, unfortunately, however, the infant is deprived of its natural food, the important inquiry presents itself, is there any alimentary substance by which the absence of that food may be supplied?

The simplest substitute for human milk would seem to be, very clearly, milk from another animal. The milk of every animal is not, however, equally adapted to supply to the human infant the want of its mother's milk:—that furnished by the different mammalia presenting a very great difference in its composition. Cow's milk is usually employed, though the milk of the ass is said, by some, to come nearer in its properties to human milk, and after that, the milk of the goat.

Dr. Routh has very clearly shown that the popular opinion once current in respect to ass's milk is incorrect. It contains certainly more water, only about half as much casein and butter, with about twice as much sugar and salts. It is believed that the purgative and sickening effects occasionally observed to follow its use are to be attributed to the excess of saline matters. Its deficiency in casein and butter may be obviated, Mr. Lobb remarks, by adding to it some two and a half per cent. of cream. In this form, he considers it to be a very good substitute for human milk, and one that could be readily procured at a comparatively trifling expense.

Goat's milk, according to the analysis of Boysson, has a composition not unlike human milk. It contains, it is true, more sugar and salts; still, it approaches much nearer to human milk than that of the ass. The best quality is furnished by the goat, we are told, about two months after kidding. The peculiar odor sometimes observed in goat's milk from the presence of hircic acid is disagreeable to many who use the article for the first time. This odor is not essential to the milk. It is chiefly present, and to the greatest extent, when the goat is allowed to associate with the ram. It is greatly diminished when the animal is kept clean, and entirely removed if it be frequently washed. The milk of the hornless goat has scarcely more odor than that of the cow.

Cow's milk is the one which in England and this country is most commonly resorted to as a substitute for human milk. From the most reliable analysis it appears that, compared with the milk of the human female, it contains less water, a greater quantity of solid matters, less sugar, and more casein and butter; it is evident, therefore, that it is but illy adapted as a substitute for the natural aliment of the human infant.

A fact, commented upon at some length by Dr. Routh, should be kept constantly in mind—namely, that the milk of all animals undergoes important modifications, according to the quality and sufficiency of food upon which the animal is fed, the manner in which it is cared for, and a number of other circumstances, all of which must be taken into consideration in deciding upon the eligibility of the milk as a substitute for that of the human female. This is especially true in respect to the milk of the cow, in the qualities of which a wide difference will be found to exist, according as it is furnished by an animal kept in town or in the country—one confined in small, damp, filthy stables, and one allowed to roam and feed at will upon rich meadows. The milk which is furnished by the animal during the summer and winter differs also. It is unnecessary to refer to the deterioration of milk as a food for infants by various adulterations, by the spontaneous changes which it undergoes when kept too long, and by the removal of the cream in part or entirely.

Among other substitutes that have been proposed for human milk, Dr. Routh enumerates particularly cream, eggs, dessicated milk, bone soups and jellies, beef-tea, and raw meat.

A mixture of one part cream and three of water will often furnish a good diet, when the milk of the cow will be found to create in the stomach a good deal of acidity. The tendency to the formation of acid may be still further obviated by adding to every half pint of the cream half an ounce of lime-water, which will have the good effect also of promoting assimilation by insuring the solubility of the casein, and the emulsion of the fatty matter.

Some of the forms of dessicated milk, when dissolved in a proper amount of water, will often furnish a good substitute for the breast-milk of the mother, or for that of a suitable and healthy nurse.

Eggs very nearly resemble milk in their composition, and form a good substitute for that of the human female. When used, the eggs should be as nearly raw as possible, or only heated to 130° Fahr. From the large amount of casein it contains, the egg should be diluted, and if, at the same time, a little sugar of milk is added, it will then form a very fair substitute for breast-milk, as the diet of a young infant.

In respect to bone soups and jellies, Dr. Routh remarks that the gelatine which is their chief ingredient, although a nitrogenous substance, is, like hair, innutritious. It overloads the blood with nitrogenous products, rendering it impure and unfitted to supply the wants of the system. As an emulcent in cases of intestinal inflammation, or a vehicle for the exhibition of wine or particular remedies, jellies may be occasionally useful.

Beef-teas, when properly prepared, form in many cases, in the absence of the breast-milk, a useful and sufficiently nutritive diet.

"If," Dr. Routh remarks, "beef-tea be prepared on Liebig's plan, and then, after a few hours' maceration in cold water, the artificial gastric juice be added, and the temperature raised to 70° Fahr., some of the fibrin will be taken up as well as the albuminous matters before in solution in the supernatant water, and a beef-tea very rich in azotized food, in fact an artificial chyme, will be obtained. For cases of defective or weak assimilation, the advantages of such a beef-tea are very obvious."

The same result may be obtained by addition of liquor pepsinii, or the essence of rennet. Dr. Routh suggests the addition to beef-tea or milk, of a solution of sweet-bread, pancreatic tea, or broken-down pieces of the pancreas itself. He believes that it would facilitate the saponifaction of fatty substances, the conversion of starch into sugar, and probably also the solution of azotized matters.

We have now the evidence of many distinguished practitioners as to the facility with which raw meat, as administered by Professor Weisse, of St. Petersburg, is digested by the infant stomach and the avidity with which it is taken. The lean of either beef or mutton, very finely shred, may be given to children of a year old, at first in portions of two teaspoonfuls four times a day, and afterward, if they crave it, the quantity may be increased. In the exhaustive diarrhœa of defective assimilation, Dr. Routh views it as his chief remedy. Meat to be given raw should be carefully examined to insure its being healthy in quality and free from parasites. If it be gray, and there are spots on its surface, it is to be suspected. A glass of small magnifying power will at once, however, remove all doubt.

Dr. Routh directs attention next to the importance of the presence of a due amount of saline ingredients in milk, and in its substitutes, in a dietetic point of view. Upon the presence in the food, in proper proportions, of phosphate of lime, especially in combination with carbonate of lime, is dependent the due solidity of the bony skeleton. Its presence, likewise, has the property of enabling the blood to take up more carbonic acid, which, when in excess, facilitates the solution by the blood of carbonate of lime, and its deposit in the bones. The presence of phosphate of soda causes the fatty acids to be converted into emulsions in the chyle, so as to admit of their ready assimilation in the system. The importance of a due amount of phosphoric acid and of chloride of potassium in the food of infants, and the important part these substances play in the functions of cell generation and of nutrition generally, is dwelt upon at some length by Dr. Routh.

While the diet of the infant, to render it well adapted to the proper support and due and symmetrical development of the infant organism, requires to be composed of a just amount of the fatty, saccharine, and caseous elements, an excess of either of them is attended with many disadvantages, which are pointed out by our author. The proper portion of butter in the food of infants he lays down as 1 part in 2·8, which is its proportion in human milk, or, taking solid constituents only, as much as 1 part in 3; of sugar, the proper proportion in milk should be 1 part in about 2·3, or, taking solid constituents, 1 in 2·6. The proportion of casein varies from 20 to 40 per cent. in 1000 parts, or, in reference to solid constituents, 1 part in 5 to 1 part in 10.

All of the several ingredients which enter into the composition of the milk of the human female must be present in whatever food is given to an infant, in order to adapt it for the proper support and development of the system. It is essential to keep this in mind in directing a course of artificial alimentation.

The next questions considered by Dr. Routh are—1, the means of correcting inferior milks, so as to adapt them for the sustenance of infants; and, 2, the preparation of a fluid to resemble human milk. In respect to the first, he refers with approbation to the plan of Dr. Merei, who uses, to dilute milk with excess of casein, a decoction of arrow-root instead of water; in this manner furnishing a substance which, by its mechanical properties, prevents to some extent the irritation of the intestinal mucous membrane. In the case of young infants cream is also to be added.

"If an infant be tolerably strong and regular in his bowels, and it has to be bottle-fed, under four months of age, a mixture of first-rate quality of milk simply with water, in equal proportions, or, after three or four months, one part of water to two of milk, agrees well, if given at a temperature of 90°. For children liable to diarrhœa, a very thin and weak infusion of anise-seed tea, instead of water, may be substituted; where the gripings and diarrhœa are severe, it is well to combine a teaspoonful, three or four times a day, of dill or peppermint-water and water in equal parts, with lime-water and a trace of opium to relieve irritation."

For the several methods furnished for the preparation of a suitable artificial milk we must refer to the work itself.

Dr. Routh discusses at great length the subject of vegetable substitutes for human milk, and proves very clearly their entire unadaptedness for the nourishment of an infant, in the early period at least of its existence. That much suffering and serious disease, and many a death among young infants, have been produced by the attempt to rear them on farinaceous preparations, we are convinced by what has fallen under our own observation. Dr. Routh would proscribe vegetable food entirely from the dietary of the infant previously to the eighth month. The organs of digestion will have reached, he believes, that degree of development at the latter period which capacitates them for digestion and assimilation of vegetable aliment. But even then he insists that only such as is most easily digestible should be at first used, and that the animal milks be given in combination.

*Part the fourth* is devoted to a consideration of the general treatment of defective assimilation.

The importance of the mother's breast-milk to secure regular and perfect assimilation in the infant has already been pointed out, and the very great difficulty to supply the want of it by the substitution of a proper wet-nurse or by hand-feeding. Dr. Routh consequently insists upon the importance of securing the maternal breast-milk to the infant as long and to as great an extent as possible. Even when unable to supply sufficient for its support, the mother should not be allowed to desist from nursing, but her deficiency of milk should be made up by giving to the infant artificial food.

Defective lactation in the female, Dr. Routh refers either to a state of hyperæmia consequent upon overfeeding, etc., a weakened or anæmic state of the body, or a torpor of the mammæ. The first cause is the least common and most easily remedied; the third is the most common. It usually occurs in females of middle age, or those who have married late in life; particularly such as are somewhat masculine in form and character. It may result from paralysis, mental emotion, disease of the sexual organs, with or without atrophy of the mammary glands, excessive obesity, exposure to impure air, or from the neglect of regular lactation. When resulting from hyperæmia, Dr. Routh directs a gentle purgative, a simple, unexciting diet, and the entire relinquishment of fermented or distilled liquors. In regulating the diet care should be observed, at the same time, that a sufficient amount of nutritious food be allowed to prevent an entire suspension of the secretion of the milk. When defective lactation is connected with debility or anæmia, the diet should be nutritious; light stimulants and tonics are to be administered, and daily exercise, with exposure to a pure free atmosphere directed. When there is complete torpor of the breasts, in many cases lactation cannot be restored; in others, however, a due secretion of milk may be induced by artificial suction, by electricity, and by a proper diet and regimen, by certain local applications, and perhaps by *galactogogues*, given internally. Among the latter, a decoction of the leaves and stalks of the *palma christi* or *ricinus communis*, and an infusion of fennel seed, are noticed as among the most reliable.

When there is no possibility of suckling the child at the mother's breast, and a suitable and trustworthy wet-nurse cannot be procured, the milk of the ass, goat, mare, or cow, must be resorted to for its food. When the milk of the cow is the food chosen, it should be obtained from a healthy animal at grass, or, during the winter season, one kept in a clean, well ventilated stable, and fed on suitable and sufficient food. The milk should be given diluted with an equal quantity or twice as much water, according to the age of the infant, the extent of the dilution being less according as the child advances in age. When the dilution is that of two parts of water to one of milk, one to two drachms of refined sugar should be added to each pint, and with every pint of diluted milk there should be mixed an ounce to an ounce and a half of lime-water, to neutralize the amount of acid that may be present, and which, if not corrected, will be productive in the infant that partakes of it of frequent hiccough, occasional crying, especially after feeding, with drawing up, perhaps, of the legs, followed by loose, greenish discharges from the bowels. When vomiting of matters having an intensely acid smell is present, the addition to the milk of lime-water in excess is indicated. The presence of flatulence and colicky pains call for the use of some light carminative; or, in severe cases wine-whey, made by adding to two parts of boiling milk one of good sherry or port wine.

The inferior kinds of milk may be corrected by the addition of arrowroot and cream, directions for which are given by Dr. Routh. In cases where obstinate diarrhœa is present, rice-water, instead of simple water, should be used as a means of dilution.

In some cases the diarrhœa is attended with great debility and extreme irritability of stomach; no kind of food is retained, even those substances which had before agreed best with the infant are rejected. In such cases Dr. Routh strongly advocates a resort to a diet of raw meat; it often, he assures us, will settle the stomach and intestines when every other form of aliment is rejected by vomiting, or passes quickly through the bowels unchanged. From ample personal experience of its good effects, he pronounces it to be one of the best and surest remedies we possess in cases of infantile atrophy from defective assimilation.

In the treatment of the atrophy of infants consequent upon defective assimilation, feculent and saccharine articles of food must be entirely proscribed. In many cases albuminous and oily substances, milk, even that of the female breast, will pass through the entire alimentary canal without having undergone much if any change. From diet in such cases little can be expected.

To remedy the defective assimilation in these cases, it has been proposed to administer artificial gastric and pancreatic juices, and the phosphate of soda. The remarks of Dr. Routh on the effects of these articles in facilitating the digestion and assimilation of albuminous food, of fatty matters, and perhaps of sugars also, are full of interest, and may be consulted with profit. The fatty acids may be given, and will be found to be readily absorbed, when the fats themselves remain unacted on. Dr. Routh thinks that the good effects of cod-liver oil in the atrophy of infants is due to its excess of fatty acid, and that the same is true in respect to butter. Children will often be found to thrive well on bread and butter when no other food is assimilated.

In the milder cases of the disease, by due attention to the diet and regimen of the infant, with the occasional use of carminatives, alkalies in small doses, and, when needed, half a drachm or so of castor oil, the cure will often be readily effected. In most cases the cod-liver oil, to the extent of a teaspoonful, with the addition, if there is much acidity, of one or more drops, according to the age of the child, of liquor potassa, will be found useful. When there is much indigestion the oil may advantageously be combined with from half a drachm to a drachm of the essence of rennet. In more severe cases, when diarrhœa is present, the best remedy, according to Dr. Routh, is the nitrate of silver in the dose of one-sixteenth to one-eighth of a grain; the sulphate of copper, in the same dose, is sometimes beneficial. All other astringents he pronounces of little avail. When the patient is nervous, over-excited, and entirely deprived of rest or sleep, anodynes become absolutely necessary. Under such circumstances Dr. Routh recommends two to five drops of henbane, in a teaspoonful of dill water at night. After a week or ten days the child will be found to rest without its use. Opium or its preparations is always to be considered a very dangerous remedy in the case of young children. A single drop of laudanum has been known to destroy life. Dr. Routh, however, remarks that when given in the dose of a quarter drop, gradually increased, and its effects carefully watched, the danger attending its use in infants may, in a great measure, if not entirely, be obviated.

When there is feverish excitement, which may come on at night, and is in a great measure the result, doubtless, of gastric irritation, Dr. Routh recommends, as the most effective remedy, external inunctions, with oily or lardaceous substances. In what manner this inunction exerts its curative influence he is unable, satisfactorily, to explain, but of the fact of such influence he has entirely satisfied himself. He assures us that, if the surface of the child be completely anointed with a mixture of suet and sweet oil, of sufficient consistence to allow it to remain on the skin, this will be found, at the end of some three hours, or perhaps sooner, to have become cool and soft, while the anorexia will have disappeared, and often there will quickly ensue a comfortable sleep. The next morning the child should be washed in a warm bath. It is said that the feverish excitement will be, in general, entirely removed after two or three inunctions, the irritation of the alimentary canal being usually, at the same time, greatly benefited. When, in connection with emaciation, there is hectic fever, inunction of the surface with cod-liver oil will often remove both emaciation and fever, and improve greatly the general condition of the patient. In cases attended with dyspepsia and great emaciation, Dr. Routh states that milk baths have been found very effective; he supposes that absorption of the fatty and nutritive matters takes place through the skin. When food cannot be taken into or retained upon the stomach, nutritive injections will sometimes prolong the patient's life long enough for the irritation of the stomach to subside, and enable suitable aliments to be given by the mouth. Even cod-liver oil, Dr. Routh remarks, will be absorbed when injected into the rectum.

The milder forms of aphthæ attendant upon defective assimilation will generally yield readily to borax dissolved in honey, weak solutions of honey and other simple remedies. The malignant or contagious form demands a more vigorous treatment. The free use of wine-whey or of wine will be in general required. As a local application, the most useful, according to the experience of Dr. Routh, is a weak solution of nitrate of silver, applied by means of a sponge, two or three times a day, or the tincture of sesquichloride of iron, from one part of the tincture to from seven to an equal proportion of water. When the throat or nasal mucous membrane is covered with aphthæ Dr. Routh uses the weaker solution, which he injects, by means of a fine syringe, down the throat or up the nostrils. In conjunction with the foregoing treatment a proper hygienic course must be instituted. The infant must be kept dry, warm, and scrupulously clean, and exposed to a pure free atmosphere. Disinfectants are to be resorted to, and every measure taken which is adapted to prevent the development of that "hospital atmosphere," which is invariably generated when many children are congregated together, and which is always injurious in its effects, but particularly so in badly constructed buildings, which are illy kept, and located in damp and unhealthy neighborhoods.

The foregoing analysis will give to our readers a tolerably correct idea of the general scope and character of the work before us. The facts and arguments of the author are calculated to produce a radical and much needed reform in the feeding of infants, and in the management of the morbid results of the errors that are so constantly committed in respect to it.

